# Technical note: Coordination and harmonization of the multi-scale, multi-model activities HTAP2, AQMEII3, and MICS-Asia3: simulations, emission inventories, boundary conditions, and model output formats

**DOI:** 10.5194/acp-17-1543-2017

**Published:** 2017-01-31

**Authors:** Stefano Galmarini, Brigitte Koffi, Efisio Solazzo, Terry Keating, Christian Hogrefe, Michael Schulz, Anna Benedictow, Jan Jurgen Griesfeller, Greet Janssens-Maenhout, Greg Carmichael, Joshua Fu, Frank Dentener

**Affiliations:** 1 European Commission, Joint Research Centre, Ispra, Italy; 2 Environmental Protection Agency, Applied Science and Education Division, National Center for Environmental Research, Office of Research and Development, Headquarters, Federal Triangle, Washington, DC 20460, USA; 3 Environmental Protection Agency, Computational Exposure Division, National Exposure Research Laboratory, Office of Research and Development, Research Triangle Park, NC 27711, USA; 4 Norwegian Meteorological Institute, Oslo, Norway; 5 Center for Global and Regional Environmental Research, University of Iowa, Iowa City, IA 52242, USA; 6 Department of Civil & Environmental Engineering, University of Tennessee, Knoxville, TN 37996, USA

## Abstract

We present an overview of the coordinated global numerical modelling experiments performed during 2012–2016 by the Task Force on Hemispheric Transport of Air Pollution (TF HTAP), the regional experiments by the Air Quality Model Evaluation International Initiative (AQMEII) over Europe and North America, and the Model Intercomparison Study for Asia (MICS-Asia). To improve model estimates of the impacts of intercontinental transport of air pollution on climate, ecosystems, and human health and to answer a set of policy-relevant questions, these three initiatives performed emission perturbation modelling experiments consistent across the global, hemispheric, and continental/regional scales. In all three initiatives, model results are extensively compared against monitoring data for a range of variables (meteorological, trace gas concentrations, and aerosol mass and composition) from different measurement platforms (ground measurements, vertical profiles, airborne measurements) collected from a number of sources. Approximately 10 to 25 modelling groups have contributed to each initiative, and model results have been managed centrally through three data hubs maintained by each initiative. Given the organizational complexity of bringing together these three initiatives to address a common set of policy-relevant questions, this publication provides the motivation for the modelling activity, the rationale for specific choices made in the model experiments, and an overview of the organizational structures for both the modelling and the measurements used and analysed in a number of modelling studies in this special issue.

## 1 Introduction

The Task Force on Hemispheric Transport of Air Pollution (TF HTAP) was organized in 2005 under the UNECE Convention on Long-range Transboundary Air Pollution (CLRTAP) (see http://www.unece.org/env/lrtap/welcome.html). Recognizing the increasing importance of hemispheric transport of air pollution, CLRTAP mandated the TF HTAP to work in partnership with scientists across the world to improve knowledge on the intercontinental or hemispheric transport and formation of air pollution; its impacts on climate, ecosystems, and human health; and the potential mitigation opportunities.

In 2010, TF HTAP produced the first comprehensive assessment of the intercontinental transport of air pollution in the Northern Hemisphere (TF HTAP, 2010a, b). A series of four reports addressed issues around emissions, transport, and impacts of particulate matter and ozone, mercury, and persistent organic pollutants (POPs), as well as their relevance for policy. The HTAP Phase 1 (HTAP1) joint modelling experiments, in which more than 20 global models participated, focussed on the meteorological year 2001. In 2012, the TF HTAP launched a new phase of cooperative multi-model experiments and analyses to provide up-to-date information to CLRTAP (e.g. Maas and Grenfellt, 2016) and other multi-lateral cooperative efforts, as well as national actions to decrease air pollution and its impacts.

The objectives of the HTAP Phase 2 (HTAP2) activity are summarized as follows:

– to estimate relative contributions of regional and extra-regional sources of air pollution in different regions of the world, by refining the source–receptor relationships derived from the HTAP Phase 1 simulations;
– to provide a basis for model evaluation and process studies to characterize the uncertainty in the estimates of regional and extra-regional contributions and understand the differences between models;
– to give input to assessments of the impacts of control strategies on the contribution of regional and extra-regional emissions sources to the exceedance of air quality standards and to impacts on human health, ecosystems, and climate.

The major advances of HTAP2 over the earlier HTAP1 experiments were the following:

– A focus on more recent years as a basis for extrapolation (2008–2010), including an updated collection of emission inventories for 2008 and 2010 (Janssens-Maenhout et al., 2015) that is utilized across all model experiments. In HTAP1 the year of interest was 2001, and in contrast to HTAP2, the anthropogenic emissions used by the different modelling groups were expected to be loosely representative for the beginning of the 2000s but were not prescribed, resulting in a large diversity of baseline emissions.
– An expanded number of more refined source/receptor regions: the original set of four rectangular (in latitude–longitude coordinates) source regions (North America, Europe, south Asia, and east Asia) identified in HTAP1 have been refined to align with geo-political borders, and additional regions have been added, dividing the world into 16 potential source regions and 60 receptor regions.
– The use of regional models and consistent boundary conditions from selected global models for Europe, North America, and Asia to provide high-resolution estimates of the impacts on health, vegetation, and climate, in addition to the global models’ worldwide coverage.

The most innovative aspect of the modelling work, performed in 2013–2016, is the consistent coupling of global and regional model experiments using existing modelling frameworks. The regional counterparts of the HTAP2 activity are the AQMEII (Air Quality Model Evaluation International Initiative) and MICS-Asia (Model Intercomparison Study for Asia) activities.

The AQMEII project was launched in 2008 in an attempt to bring together modellers from both sides of the Atlantic Ocean to perform joint regional model experiments using common boundary conditions, emissions, and model evaluation frameworks with a specific focus on regional modelling domains over Europe and North America (Rao et al., 2012). The first two AQMEII activities focused on the development of general model-to-model and model-to-observation evaluation methodologies (phase 1; Galmarini et al., 2012a) and the simulation of aerosol–climate feedbacks with online coupled modelling systems (phase 2; Galmarini et al., 2015). AQMEII Phase 3 (AQMEII3) is devoted to performing joint modelling experiments with HTAP2. The AQMEII modelling community (Table 5) includes almost all of the major existing modelling systems for regional-scale chemical transport simulation in research and regulatory applications on both continents. Most of the groups participating are part of modelling initiatives in the individual European member states, and some of these groups utilize models developed in North America, thus providing the opportunity of assessing the application of these models outside of their conventional modelling context.

The MICS-Asia Phase III (MICS3) project is an activity building on work performed in phase I (1998–2000; sulfur transport and deposition) and phase II (2004–2009; sulfur, nitrogen, ozone, and aerosols; see Fu et al., 2008). MICS3 is organized as a multi-national consortium of institutions and brings together modellers from China, Japan, Korea, southeast Asia, and the United States (Table 6). The overall scope of MICS3 includes evaluation of the ability of models to reproduce pollutant concentrations under highly polluted conditions, dry and wet deposition fluxes, and the quantification of the effects of uncertainties due to process representation (emissions, chemical mechanisms, transport and deposition) and model resolution on simulated air quality. The joint evaluation with HTAP2 focuses on the evaluation of the role of long-range transport of air pollution both within and to/from east Asia on air quality and impacts on climate, ecosystems and human health.

The framework used for global aerosol modelling is the AeroCom initiative (Aerosol Comparison between observations and models; Schulz et al., 2009; Myhre et al., 2013), and dedicated experiments on long-range transport were designed and performed in collaboration with HTAP as part of AeroCom phase 3 (see https://wiki.met.no/aerocom/phase3-experiments), with an additional focus on long-range transport of dust and fire-derived aerosol. The data storage and evaluation platform for global models was shared between AeroCom and HTAP2 (see Sect. 2.5).

Presently these three activities involve 23 global-scale models (Table 3) and approximately 30 regional-scale modelling groups performing model simulations in the North American, European, and east Asian domains, probably making the HTAP2–AQMEII3–MICS3 exercise the largest multi-scale/multi-model activity ever performed in atmospheric chemical modelling. The multi-scale and multiregional modelling exercise required three independent organizations to manage and engage their respective communities and an overarching coordination effort as well as a high level of harmonization of the model simulations aiming at comparability, usability, and interoperability of the model results at the various scales. Specific decisions were made regarding the simulation period, lower air boundary conditions (emission inventory), volatile organic compound (VOC) speciation, methane concentrations, emission perturbation runs, source region perturbations, lateral and upper air boundary conditions for regional simulations, variables expected for the analysis, file naming conventions, type and location of monitoring sites where model results were output, data submission procedures, and the development and use of interoperable data archiving and visualization servers.

The scope of this note is to provide information on the modelling activity harmonization and coordination adopted to guarantee the maximum level of coherence between the global and regional simulations. It provides specific details on the organization of the global HTAP2 and the regional AQMEII3 activities, but only general information on the MICS3 experiments is provided. Additional details regarding HTAP2 are summarized at http://iek8wikis.iek.fz-juelich.de/HTAPWiki/ and are available in the report by Koffi et al. (2016) and for AQMEII3 at http://ensemble2.jrc.ec.europa.eu/aqmeii/.

This note provides coherent information on the simulations performed and their characteristics to support the analysis articles presented in this special issue.

## 2 The HTAP2, AQMEII3, and MICS3 modelling exercises set-up

The following aspects were harmonized in the organization of this multi-scale, multi-chemical-transport-model activity:

– Simulation periods and meteorology to be used.
– Emission inventories for global and regional models.
– Boundary conditions for regional-scale air quality models.
– Harmonization of global and regional model output and interoperability of data repositories to facilitate the exchange and analysis of model outputs.
– Monitoring data locations and methods for comparing models with observations.
– Documentation of individual model set-up and construction of ensemble averages.

### 2.1 Simulation period and meteorology used

The simulation period of interest 2008–2010 was chosen on the basis of the availability of emissions data and intensive observations. The models were requested to run the 3-year period with a priority given to the year 2010, followed by 2008 and then 2009. Global models can use meteorological data representative of the respective year, e.g. driven or constrained by one of the global analysis products that were most convenient to the modelling group. Regional-scale modellers also were free to use the meteorological model of their choice based on compatibility with their chemical transport model. Sets of chemical boundary conditions for the regional models were provided by a limited set of global models participating in the global modelling experiments (see Sect. 2.4)

### 2.2 Emission data

The anthropogenic emission data were harmonized across the regional and global modelling experiments. The Joint Research Centre’s (JRC) EDGAR (Emission Database for Global Atmospheric Research) team – in collaboration with regional emission experts from the US Environmental Protection Agency (EPA), EMEP (European Monitoring and Evaluation Programme), CEIP (Centre on Emission Inventories and Projections), TNO (Netherlands Organisation for Applied Research), the MICS-Asia scientific community, and REAS (Regional Emission Activity Asia) – has compiled a composite of regional emission inventories with monthly grid maps that include EDGARv4.3 gap filling for regions and/or sectors that were not provided by the regional inventories.

The HTAP_v2.2 database (Janssens-Maenhout et al., 2015), used in the global modelling experiments, has the following characteristics:

– years 2008 and 2010, yearly and monthly time resolutions;
– components: SO_2_, NO_*x*_, non-methane VOC (NMVOC), CH_4_, CO, NH_3_, PM_10_, PM_2.5_, BC, and OC at sector-specific level;
– seven emission sectors (Janssens-Maenhout et al., 2015); see Table 1;
– global coverage with spatial resolution of 0.1° × 0.1° longitude and latitude, to serve the needs of both global and regional model activities.

Annual gridded emission data (http://edgar.jrc.ec.europa.eu/htap_v2) are delivered for each pollutant and emission sector. Monthly gridded values are provided for some sectors (energy, industry, transport, and residential), where information was available to disaggregate annual emissions. For 2009 no emissions were provided, leaving the choice to the modelling group to either interpolate the 2008 and 2010 data or leave them constant.

**Figure 1:**
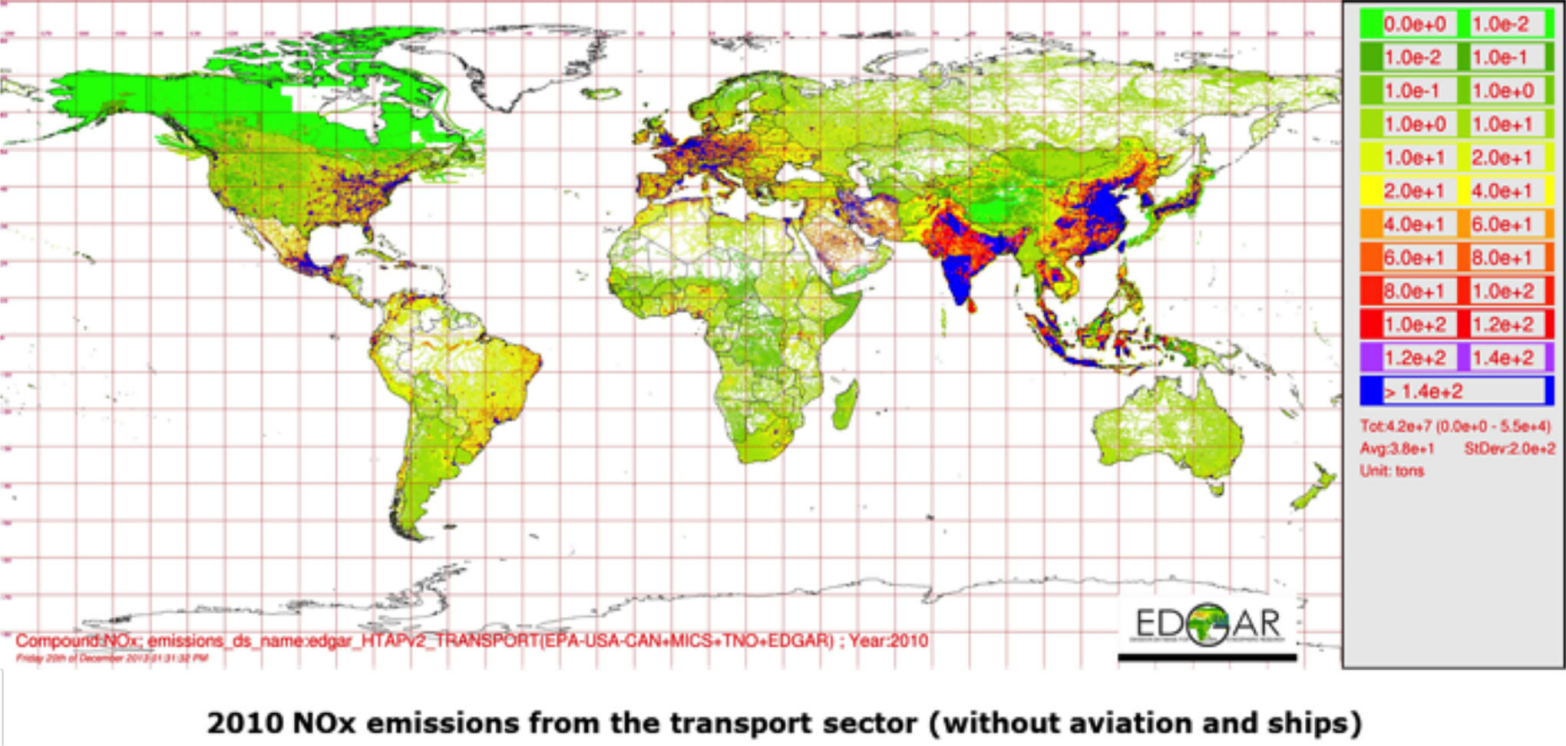
Example of HTAP_v2.2 emission mosaics for NO_*x*_ in the transport sector.

The regional emissions for the North American and European regional-scale simulations of AQMEII3 are described in Pouliot et al. (2015), were used earlier for AQMEII2 (Galmarini et al., 2015), and are embedded into the HTAP_v2.2 inventory. The Asian inventory MIX (Li et al., 2015) was developed for MICS3 and HTAP2 simulations on a 0.25° × 0.25° resolution and converted by raster resampling to 0.1° × 0.1° resolution for use in HTAP2. These regional inventories have been combined to form a global mosaic (Fig. 1) that is consistent with inventories used at the regional scale in Europe, North America, and Asia. However, we note that these emission estimates stemming from different data sources for different regions of the world are not necessarily consistent with each other; for example different fuel statistics or emission factors may have been used for different regions. Details on the recommended VOC speciation and other specific emission information can be found in Koffi et al. (2016), Janssens-Maenhout (2015), Li et al. (2015), and Pouliot et al. (2015).

Biomass burning emissions have not been prescribed for the global modelling groups, but it is recommended that groups use Global Fire Emission Database version 3 (GFED3) data, which are available at daily and 3 h intervals (see http://globalfiredata.org/). For the regional modelling groups participating in AQMEII3, fire emissions were included in the inventories distributed to the participants (Pouliot et al., 2015; Soares et al., 2015). Biogenic NMVOCs, soil and lightning NO_*x*_, dust, and sea salt emissions have not been prescribed for either the global or regional modelling groups; modelling groups are encouraged to use the best information that they have available, except that the AQMEII3 regional modelling groups were advised not to include lightning NO_*x*_ in their simulations since not all modelling groups had a mechanism for including them. For wind-driven DMS (dimethyl sulfide) emissions from oceans, the climatology of ocean surface concentrations described in Lana et al. (2011) was recommended in conjunction with the model’s meteorology and emission parameterization for the global models. The regional models participating in AQMEII3 did not consider DMS emissions. For volcanic emissions, it was recommended that global groups use the estimates developed for 2008–2010 for AeroCom as an update of the volcanic SO_2_ inventory of Diehl et al. (2012) and accessible at http://aerocom.met.no/download/emissions/HTAP/. As in the case of lightning NO_*x*_ emissions, the AQMEII3 regional modelling groups were advised not to include volcanic emissions in their simulations since not all modelling groups had a mechanism for including them. Modelling groups were asked to document the source of all of their emissions data and assumptions, especially if it deviated from the recommended parameterizations. For mercury, the Arctic Monitoring and Assessment Programme (AMAP)/United Nations Environmental Programme (UNEP) global emissions inventory for 2010 was recommended (http://www.amap.no/mercury-emissions). None of the regional models participating in AQMEII3 considered mercury in their simulations.

### 2.3 Emission perturbation

**Figure 2:**
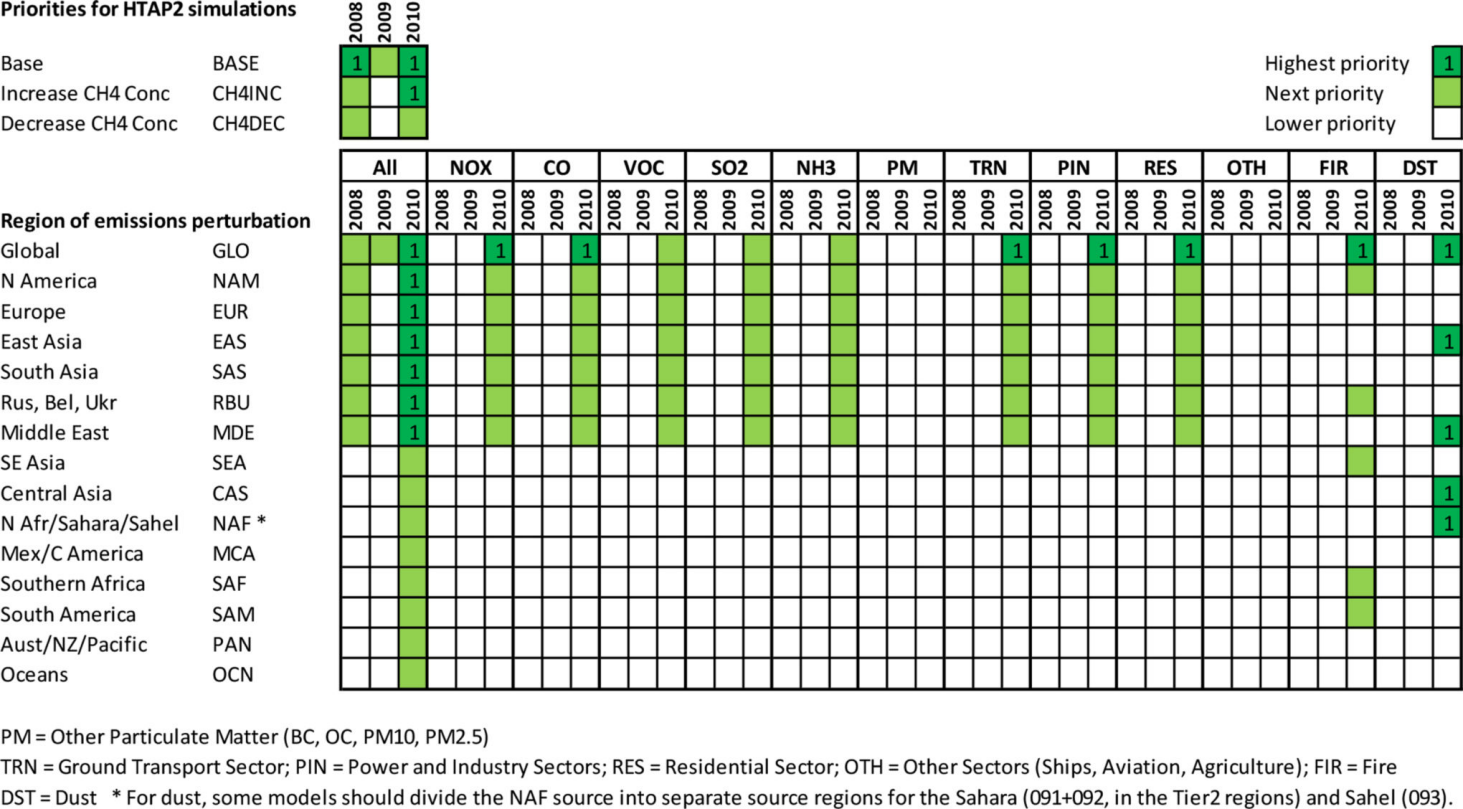
HTAP2 emission perturbation experiments; dark green colour with 1 are highest priority experiments, light green next priority, and white lower priority. ALL refers to perturbation of all anthropogenic components and sectors; sectors are TRN (transportation), PIN (power + industry), RES (residential), OTH (Other), FIR (fire), and DST (mineral dust).

In addition to the base 2008–2010 simulations, modelling groups were requested to perform emission perturbation experiments to help estimate source–receptor relationships; to attribute estimated concentrations, deposition fluxes, and derived impacts to regional and extra-regional sources; and to be used for scenario evaluations including uncertainties. Fig. 2 lists a large number of possible perturbation experiments; all except the methane perturbation experiments involve a 20% decrease in anthropogenic emissions similar to HTAP1. The choice of 20% was motivated by the consideration that the perturbation would be large enough to produce a sizeable impact (i.e. more than numerical noise) even at long distances while small enough to be in the near-linear atmospheric chemistry regime, assumptions which are subject to further analysis. The emission decreases are specified for combinations of pollutants, regions, and sectors.

To capture the impact of changing methane emissions in a single-year simulation, it is necessary to perturb the methane concentration instead of the emissions. The recommended perturbations (Table 2) are intended to cover the range of CH_4_ concentration changes associated with the Representative Concentration Pathway (RCP) scenarios used for the Intergovernmental Panel on Climate Change (IPCC) Fifth Assessment Report (AR5) (IPCC, 2013) for 2030. The highest priority was assigned to an increase of global CH_4_ concentrations to 2121 ppbv (representative of RCP8.5). The next priority is assigned to a decrease of global CH_4_ concentrations to 1562 ppbv (representative of RCP2.6).

The combination of global (all regions and sources) and regional perturbation experiments provides the necessary information to calculate the so-called RERER (response to extra-regional emission reductions) metric, using the information on the contribution of foreign emission perturbations relative to all worldwide emission perturbation to a change in region *i*.

(1) $${\text{RERER}}_{i}=\frac{\sum R_{\text{foreign}}}{\sum R_{\text{all}}}=\frac{R_{\text{global}}-R_{\text{region},i}}{R_{\text{global}}},$$

where *R*_global_ is the global response of a quantity (e.g. surface O_3_ concentration) in the global 20% perturbation simulation (GLO) minus the value in the unperturbed simulation (BASE) and *R*_region_ is the regional response of that quantity in the regional 20% emission perturbation simulation minus its value in BASE. The metric can be applied to a range of quantities, including surface concentrations, column amounts, and derived parameters.

A low (i.e. near 0) RERER value means that the signal within a region is not very sensitive to extra-regional emission reductions and that local concentrations (or column amounts, etc.) depend more on local emission reductions given the current distribution of anthropogenic and biogenic emissions. A high RERER value (i.e. near 1) suggests that local conditions are strongly influenced by emissions changes outside the region. In some circumstances, when emission reductions correspond to increasing concentrations (e.g. ozone titration by NO emissions), RERER can be larger than 1.

### 2.4 Boundary conditions for regional simulations

One of the new aspects of HTAP2 experiments is the coupling of global and regional model simulations, including coupled emission perturbation studies. These common experiments are intended to enable examination of the effects of (a) finer spatial and temporal resolution of regional models and (b) different processes represented in global and regional models.

**Figure 3:**
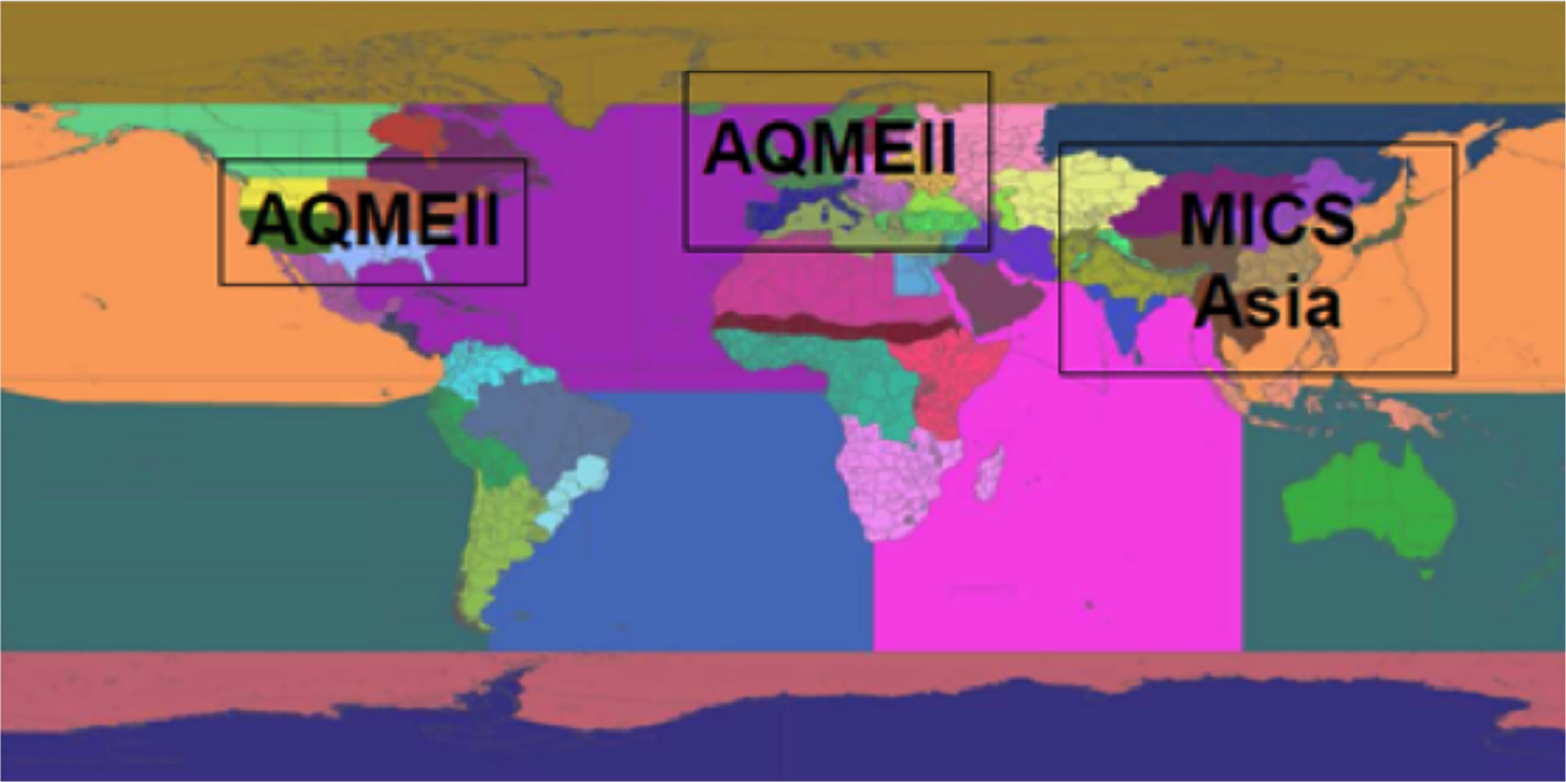
Domains of the regional model simulations and source/receptor areas.

In order to “nest” the regional within the global simulations, computational results from one or more global models are needed as boundary conditions for the regional models’ domains (Fig. 3), typically provided as a set of time-varying concentrations of medium-to-long-lived components in a 3-D box over the respective regional model domains at typical time resolutions of 3 to 6 h.

**Figure 4:**
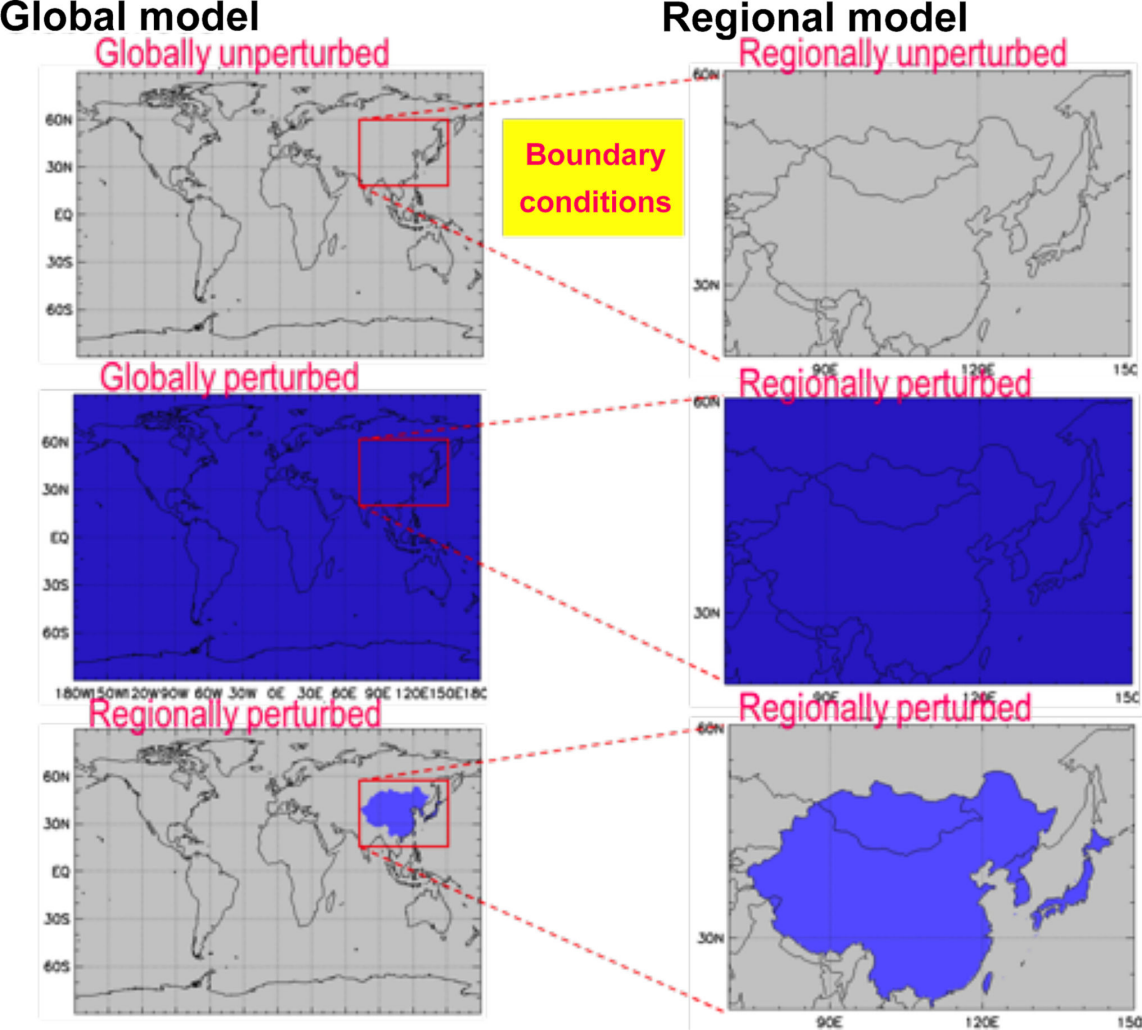
Example set of experiments, with both a global and regional model (in this case a regional model over east Asia, red box), where the regional source perturbation is east Asia (blue shading) and is wholly within the regional model domain. Note that the magnitude of the emission perturbation in the region of consideration is identical between the global and regional model.

A small number of the global models participating in HTAP2 provided boundary conditions for regional simulations, the choice depending mostly on existing experiences of regional communities with these particular global models. The global-scale simulations that were made available to the regional-scale modellers for defining boundary conditions are presented in Table 3. Boundary conditions were provided both for the BASE case and for a number of emission perturbation runs. Each of the emissions perturbation experiments with the global models created a new set of boundary conditions that can be used at the regional scale. This nesting is depicted graphically in Fig. 4. It shows an example where the HTAP2 source region (in this case, east Asia) is wholly within the regional model domain. The inclusion of the global perturbation simulation (GLO scenario for all pollutants: GLOALL) allows consistent evaluation of the RERER metric for the 20% reductions of all emissions in both global and regional models (see Sect. 2.3).

Regional models were free to use as boundary conditions one or more models as long as they were selected from the set of global models participating in HTAP2, but in practice the AQMEII3 community focused its effort on C-IFS(CB05) (Flemming et al., 2015) calculations. Geophysical Fluid Dynamic Laboratory/Atmospheric Model 3 (GFDL/AM3; Lin et al., 2012a, b) and the Global Earth Observing System Chemistry model (GEOS-Chem; Park et al., 2004; Bey et al., 2001) were additionally used in some North American simulations. GEOS-Chem and CHASER (Sudo et al., 2002; Sudo and Akimoto, 2007; Watanabe et al., 2011; Sekiya and Sudo, 2014) were the preferred models for the MICS3 consortium.

### 2.5 Specification of the global- and regional-scale model outputs

Careful consideration was given to the organization of the model output, given the large number of models, variables requested, and case studies. This required specifications of data formats, variable and file naming conventions, data organization at identified collection points, and the definition of agreed locations where measurements would be available and model data had to be produced for both regional and global models. Further details can be found at http://iek8wikis.iek.fz-juelich.de/HTAPWiki/HTAP-2-data-submission and in Koffi et al. (2016). For HTAP2 and AQMEII3, the experience acquired over the past experiments allowed this massive data handling task to be carried out in an efficient way because data formats, naming conventions, and collections points were already well established for these two activities and respective communities of models. For HTAP2 the Network Common Data Form (netCDF; http://www.unidata.ucar.edu/software/netcdf/) with Climate and Forecast (CF) (http://cfconventions.org/) meta-data format was adopted. For AQMEII3 the ENSEMBLE data format was used (Galmarini et al., 2012b), allowing easy participation for regional modellers already participating in AQMEII2. Two data repositories were available for the two communities: the AeroCom repository at the Norwegian Meteorological Institute (MetNo) (aerocom.met.no; Schulz et al., 2009) and the JRC ENSEMBLE (Galmarini et al., 2014) platforms. Data for MICS3 modelling community were handled and analysed at the Joint International Center on Air Quality Modeling Studies (JICAM) in Beijing, China, a joint cooperation between the Institute of Atmospheric Physics (IAP) of the Chinese Academy of Sciences and the Asia Center for Air Pollution Research (ACAP) in Niigata, Japan. These facilities allow not only the organization of the data produced by various sources around the world but also their consultation through web interfaces and the matching of the model results with the available measured data and the statistical comparison of these two pieces of information. A connection and automatic data conversion protocol between the ENSEMBLE and AeroCom platforms was also pioneered to allow the bidirectional transfer of model data and a consistent comparison of global and regional model results with a common set of observations.

Global model data from this study can be accessed via the AeroCom data server at MetNo. Data are organized such that the HTAP2 model version, experiment, period, and variable name can be identified readily from directory and file names. Model output providers have to register at the database provider MetNo and are provided with access to a Linux server via ssh (see further details at https://wiki.met.no/aerocom/user-server). This server also provides essential and standard data inspection, analysis, and extraction tools for netCDF files (ncdump, ncview, python, nco, cdo, etc.). Users may utilize these tools to retrieve files or subsets of them for further analysis. All incoming files are processed with the AeroCom visualization tools to generate “quicklook” images for initial inspection. All variables are plotted as fields for major regions, each month and season. Where available, comparisons are made to surface observations, mainly those from the EBAS database maintained by the Norwegian Institute for Air Research (NILU, ebas.nilu.no) and from Aeronet (http://aeronet.gsfc.nasa.gov). The quicklook images are publicly available via the web interface at http://aerocom.met.no/cgi-bin/aerocom/surfobs_annualrs.pl?PROJECT=HTAP&MODELLIST=HTAP-phaseII-ALL.

**Figure 5:**
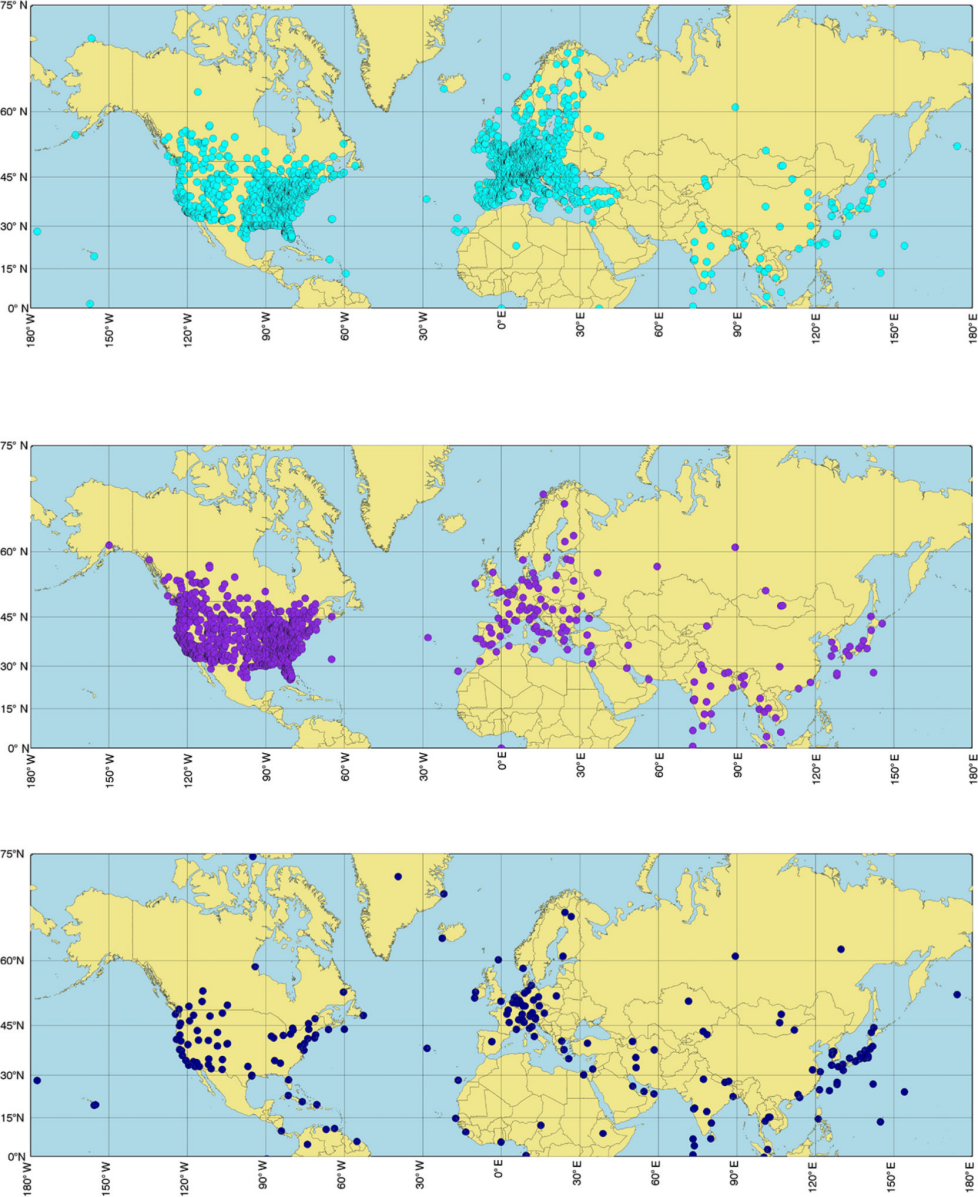
Location of the stations where surface gas (top), surface aerosol (middle), and vertical profile (bottom) model outputs are requested.

To facilitate the comparability of model results with measured data, the former were requested as time series at surface locations, or vertical profiles, mostly located in Europe and North America, enabling the comparison of the AQMEII3 and HTAP2 experiments. Model results were requested in various forms. Specifically, 4128 surface stations were identified for the comparison of gas phase species, 2068 surface stations were identified for the comparison of aerosol species, and 240 stations were identified for the evaluation of vertical profiles. These locations are a mixture of stations of global and regional significance and spatial representativeness (Fig. 5). Details of the data requests for HTAP2 can be found in Koffi et al. (2016).

For AQMEII3, the specifications of requested model variables are contained in the so-called AQMEII overarching document (http://ensemble2.jrc.ec.europa.eu/aqmeii/?page_id=527). Model results are also available to participating modelling groups and the wider scientific community through the ENSEMBLE web-based platform following the protocol established for phase 1 and 2 of AQMEII (Galmarini and Rao, 2011).

MICS3 output includes monthly averaged hourly surface data for O_3_, NO, NO_2_, HNO_3_, and HONO; surface VOC species consistent with the CB05, CBMZ, RADM2, and SAPRC99 mechanisms; and wet/dry depositions of sulfur and nitrogen components.

To help diagnose the differences between models and isolate different transport processes, we requested that HTAP2 global models also include two passive tracers. These tracers should be emitted in the same quantity as total anthropogenic CO emissions (not including fires) and decay exponentially with uniform fixed mean lifetimes (or e-folding times) of 25 and 50 days, respectively, as in the Chemistry-Climate Modelling Initiative (CCMI).

## 3 Conclusions

This technical note provides details about the set-up of the joint regional-global chemistry–transport emission perturbation experiments, planned and executed within the HTAP2 model exercise. The Task Force Hemispheric Transport Air Pollution falls under the Cooperative Programme for Monitoring and Evaluation of the Long-range Transmission of Air Pollutants in Europe (EMEP) of the UNECE Convention and is an increasingly important issue of hemispheric transport of air pollution. TF HTAP works in partnership with scientists across the world to improve our understanding of the intercontinental or hemispheric transport and formation of air pollution; its impacts on climate, ecosystems, and human health; and the potential mitigation opportunities. The major advances of HTAP2 with respect to previous HTAP1 activity are

– a focus on more recent years as a basis for extrapolation (2008–2010);
– a larger number of source/receptor regions;
– in collaboration with the existing regional-scale modelling initiatives AQMEII and MICS-ASIA, the use of regional models and consistent boundary conditions from selected global models for Europe, North America, and Asia to provide higher-resolution estimates of the impacts of hemispheric transport of air pollution on health, ecosystems, and climate.

The multi-model, multi-scale, and multi-pollutant character of the activities performed in HTAP2 required a considerable level of harmonization of the information used to run the models at different scales and of the results produced. Such harmonization considerably facilitates the interpretation of model results and inter-model differences. Particular attention was given to providing coherent emissions and boundary conditions to the global- and regional-scale models, and harmonizing monitoring data collected to evaluate the model results. To our knowledge such an attempt is unprecedented in the field and constitutes an important starting point for future multiple-scale modelling activities. A considerable effort has been made for the harmonization of data formats and web-based data hubs, allowing consultation of model and measurement data by the participants as well as possible external data users with simplicity and having a few “one-stop shops”, where all information is collected, geo-referenced, and ready to be used. As independently demonstrated in the past, by the ENSEMBLE and AeroCom experiences, such an approach effectively takes away the burden on individual modelling groups of collecting scattered measurement data and organizing these data sets for comparison with models. Moreover, this approach effectively provides benchmark data sets for objective comparisons across models.

While first steps towards fuller integration of protocols, requested outputs, and analysis methods were shared across the three communities, a fully interoperable and harmonized set of global and regional outputs was not yet obtained due to different requirements of the communities. Data can now be converted into two of the three formats available very easily (HTAP ⇔ AQMEII), and therefore the most important step to allow a full consultability of the data by the two communities has been made. The technical aspect of making the systems AeroCom and ENSEMBLE be mirrored into one another will also be explored in conjunction with available resources. All relevant elements are in place to make such steps possible. Such steps will also be performed possibly with the MICS-Asia data and information. At this stage, the availability of global and regional model outputs and observations at a common set of monitors permits a first analysis of global-regional model performance in the North American, European, and Asian domains and represents a significant step forward for both communities.

Many of the analyses presented in this special issue draw upon this unique collection of data and tools, which is open and available for further analysis. We encourage the scientific community to continue to explore these data to generate scientific and policy-relevant insights and to engage in the future development of the TF HTAP, AQMEII, and MICS-Asia activities.

## 4 Data availability

The data generated for the HTAP and AQMEII MICs-ASIA exercise are accessible through the data platforms described above upon contact with the managing organizations.

## Figures and Tables

**Table 1:** Emission sectors in HTAP_v2.2 database.

Sector	Description
Air	International and domestic aviation
Ships	International shipping
Energy	Power generation
Industry	Industrial non-power large-scale combustion emissions and emissions of industrial processes and product use including solvents

Transport	Ground transport by road, railway, inland waterways, pipeline, and other ground transport of mobile machinery; does not include re-suspension of dust from pavements or tyre and brake wear

Residential	Small-scale combustion, including heating, cooling, lighting, cooking, and auxiliary engines to equip residential and commercial buildings, service institutes, and agricultural facilities and fisheries; solid waste (landfills/incineration) and wastewater treatment

Agriculture	Agricultural emissions from livestock and crop cultivation but not from agricultural waste burning and not including savannah burning

**Table 2:** BASE and methane perturbation runs.

Simulation	Global CH_4_ concentration (ppbv)	Representative of
BASE	1798	2010 based on IPCC (2013)
CH4INC	2121	2030 under RCP 8.5
CH4DEC	1562	2030 under RCP2.6

**Table 3:** Global models and institutions participating in HTAP2.

Group/institution	Model
CICERO	OsloCTM3.v2
NASA GSFC	GOCARTv5
RIAM	SPRINTARS
NAGOYA,JAMSTEC,NIES	CHASER_re1
NAGOYA,JAMSTEC,NIES	CHASER_t106
Univ.Col. Boulder	GEOS-Chem-ADJOINT
SSEC-NESDIS	RAQMS
SSEC_NESDIS	RAQMS_ASSIM
NASA GSFC	GEOS5
GEORGIA TECH	REAM
SNU	GEOS-Chem
SNU	GEOS_Chem_Calnex
UNIMOD	EMEP_rv4.5
UNIMOD	EMEP_rv4.8
ECMWF	C-IFS
IITM	MOZART-4
UTK	HCMAQ
NCAR	CAM-chem
Environment and Climate Canada	GEMMACH
UK Met Office	HadGEM2-ES
Iowa/JPL/GMU	STEM-CIFS
Iowa/JPL/GMU	STEM-GC
Iowa/JPL/GMU	STEM-RAQMS

**Table 4:** 2008, 2009, and 2010 HTAP2 global runs for regional boundary conditions.

Model	Spatial resolution	Temporal resolution	Chemistry	Simulations
C-IFS(CB05) (ECMWF)	1.125° ×1.125°	3 hourly	CB05	BASE
	(T159)			GLOALL
	54 levels			CH4INC
				NAMALL
				EURALL
				EASALL
				SASALL
GFDL/AM3	~1° × 1°	3 hourly		BASE
	48 levels			GLOALL
				CH4INC
				NAMALL
				EURALL
				EASALL
GEOS-Chem	2.5° × 2°	3 hourly		BASE
	47 levels			GLOALL
				CH4INC
				NAMALL
				EURALL
				EASALL
CHASER	2.8° × 2.8°	3 hourly + daily mean		BASE

**Table 5:** Institutions and models involved in AQMEII.

Operated by	RCM	Emission	Horiz. Res. Lat × lon	Global Met	Chem Mod
Finnish Meteorological	ECMWF-	EDGAR-HTAP;	0.25 × 0.25^°^	ECMWF	CBM-IV
Institute	SILAM_H, SILAM_M	TNO-MACC			
Netherlands Organisation for Applied Scientific Research	ECMWF-L.- EUROS	TNO-MACC	0.5 × 0.25^°^	Interpolation from ECMWF	CBM-IV
INERIS/CIEMAT	ECMWF-Chimere_H Chimere_M	EDGAR-HTAP; TNO-MACC	0.25 × 0.25^°^	Interpolation from ECMWF	MELCHIOR2
University of L'Aquila	WRF-WRF/Chem1	TNO-MACC	23 km	ECMWF	RACM-ESRL
University of Murcia	WRF-WRF/Chem2	TNO-MACC	23 km × 23 km	ECMWF	RADM2
Ricerca Sistema Energetico	WRF-CAMx	TNO-MACC	23 km × 23 km	ECMWF	CB05
University of Aarhus	WRF-DEHM	EDGAR HTAP	50 km × 50 km	ECMWF	Brandt et al. (2012)
Istanbul Technical University	WRF-CMAQ1	TNO-MACC	30 km × 30 km	NCEP	CB05
Kings College	WRF-CMAQ4	TNO-MACC	15 km × 15 km	NCEP	CB05
Ricardo E&E	WRF-CMAQ2	TNO-MACC	30 km × 30 km	NCEP	CB05-TUCL
Helmholtz-Zentrum Geesthacht	CCLM-CMAQ	EDGAR-HTAP	24 km × 24 km	NCEP	CB05-TUCL
University of Hertfordshire	WRF-CMAQ3	TNO-MACC	18 km × 18 km	ECMWF	CB05-TUCL
Helmholtz-Zentrum Geesthacht	CCLM-CMAQ	SMOKE	24 km × 24 km	NCEP	CB05-TUCL
Environmental Protection Agency of the USA	WRF-CMAQ	SMOKE	12 km × 12 km	NCEP (nudging)	CB05-TUCL
RAMBOLL Environ	WRF-CAMx	SMOKE	12 km × 12 km	NCEP	CB05
University of Aarhus	WRF-DEHM	EDGAR-HTAP	50 km × 50 km	interpolation from ECMWF	Brandt et al. (2012)

**Table 6:** Institutions and models involved in MICS-Asia.

Group/institution	Models
National Institute for Environmental Studies, Japan	CMAQv4.7.1
Central Research Institute of Electric Power Industry, Japan	CMAQv4.7.1
Kobe University, Japan	CMAQv4.7.1
The University of Tennessee, Knoxville, USA	CMAQv5.0.2
Sun Yat-Sen University, China (SYSU)	CMAQv5.0.2
Institute of Atmospheric Physics, Chinese Academy of Sciences, China	GEOS-Chem
Institute of Atmospheric Physics, Chinese Academy of Sciences, China	NAQPMS
Meteorological Research Institute, Japan	NHM-Chem
Pusan National University, Korea (not in the analyses)	WRF-Chem
Academia Sinica, Taiwan (not in the analyses)	WRF-Chem
Institute of Atmospheric Physics, Chinese Academy of Sciences, China	RAMSCMAQ
Institute of Atmospheric Physics, Chinese Academy of Sciences, China (not in the analyses)	WRF-Chem

## References

[R1] Bey I, Jacob DJ, Yantosca RM, Logan JA, Field BD, Fiore AM, Li Q, Liu HY, Mickley LJ, Schultz MG (2001). Global modeling of tropospheric chemistry with assimilated meteorology: model description and evaluation. J. Geophys. Res.-Atmos.

[R2] Brandt J, Silver JD, Frohn LM, Geels C, Gross A, Hansen AB, Hansen KM, Hedegaard GB, Skjøth CA, Villadsen H, Zare A, Christensen JH (2012). An integrated model study for Europe and North America using the Danish Eulerian Hemispheric Model with focus on intercontinental transport. Atmos. Environ.

[R3] Diehl T, Heil A, Chin M, Pan X, Streets D, Schultz M, Kinne S (2012). Anthropogenic, biomass burning, and volcanic emissions of black carbon, organic carbon, and SO_2_ from 1980 to 2010 for hindcast model experiments. Atmos. Chem. Phys. Discuss.

[R4] Flemming J, Huijnen V, Arteta J, Bechtold P, Beljaars A, Blechschmidt A-M, Diamantakis M, Engelen RJ, Gaudel A, Inness A, Jones L, Josse B, Katragkou E, Marecal V, Peuch V-H, Richter A, Schultz MG, Stein O, Tsikerdekis A (2015). Tropospheric chemistry in the Integrated Forecasting System of ECMWF. Geosci. Model Dev.

[R5] Fu JS, Jang CJ, Streets DG, Li Z, Kwok R, Park R, Han Z (2008). MICS-Asia II: Modeling gaseous pollutants and evaluating an advanced modeling system over East Asia. Atmos. Environ.

[R6] Galmarini S, Rao ST (2011). The AQMEII two-continent Regional Air Quality Model evaluation study: Fueling ideas with unprecedented data. Atmos. Environ.

[R7] Galmarini S, Rao ST, Steyn DG (2012a). Preface to the AQMEII p1 Special issue. Atmos. Environ.

[R8] Galmarini S, Bianconi R, Appel W, Solazzo E, Mosca S, Grossi P, Moran M, Schere K, Rao ST (2012b). ENSEMBLE and AMET: Two systems and approaches to a harmonized, simplified and efficient facility for air quality models development and evaluation. Atmos. Environ.

[R9] Galmarini S, Hogrefe C, Brunner D, Makar P, Baklanov A (2015). Preface to the AQMEII p2 Special issue. Atmos. Environ.

[R10] Stocker TF, Qin D, Plattner G-K, Tignor M, Allen SK, Boschung J, Nauels A, Xia Y, Bex V, Midgley PM, IPCC, Climate Change 2013 (2013). The Physical Science Basis. Contribution of Working Group I to the Fifth Assessment Report of the Intergovernmental Panel on Climate Change.

[R11] Janssens-Maenhout G, Crippa M, Guizzardi D, Dentener F, Muntean M, Pouliot G, Keating T, Zhang Q, Kurokawa J, Wankmüller R, Denier van der Gon H, Kuenen JJP, Klimont Z, Frost G, Darras S, Koffi B, Li M (2015). HTAP_v2.2: a mosaic of regional and global emission grid maps for 2008 and 2010 to study hemispheric transport of air pollution. Atmos. Chem. Phys.

[R12] Koffi B, Dentener F, Janssens-Maenhout G, Guizzardi D, Crippa M, Diehl T, Galmarini S, Solazzo E (2016). Hemispheric Transport Air Pollution (HTAP): Specification of the HTAP2 experiments – Ensuring harmonized modelling, EUR 28255 EN – Scientific and Technical Research Reports.

[R13] Lana A, Bell TG, Simó R, Vallina SM, Ballabrera-Poy J, Kettle AJ, Dachs J, Bopp L, Saltzman ES, Stefels J, Johnson JE, Liss PS (2011). An updated climatology of surface dimethylsulfide concentrations and emission fluxes in the global ocean. Global Biogeochem. Cy.

[R14] Li M, Zhang Q, Kurokawa J, Woo J-H, He KB, Lu Z, Ohara T, Song Y, Streets DG, Carmichael GR, Cheng YF, Hong CP, Huo H, Jiang XJ, Kang SC, Liu F, Su H, Zheng B, MIX (2015). a mosaic Asian anthropogenic emission inventory for the MICS-Asia and the HTAP projects. Atmos. Chem. Phys. Discuss.

[R15] Lin MY, Fiore M, Horowitz LW, Cooper OR, Naik V, Holloway J, Johnson BJ, Middlebrook AM, Oltmans SJ, Pollack IB, Ryerson TB, Warner JX, Wiedinmyer C, Wilson J, Wyman B (2012a). Transport of Asian ozone pollution into surface air over the western United States in spring. J. Geophys. Res.-Atmos.

[R16] Lin M, Fiore AM, Cooper OR, Horowitz LW, Langford AO, Levy H, Johnson BJ, Naik V, Oltmans SJ, Senff CJ (2012b). Springtime high surface ozone events over the western United States: Quantifying the role of stratospheric intrusions. J. Geophys. Res.

[R17] Maas R, Grennfelt P (2016). EMEP Steering Body and Working Group on Effects of the Convention on Long-Range Transboundary Air Pollution. Towards Cleaner Air, Scientific Assessment Report.

[R18] Myhre G, Samset BH, Schulz M, Balkanski Y, Bauer S, Berntsen TK, Bian H, Bellouin N, Chin M, Diehl T, Easter RC, Feichter J, Ghan SJ, Hauglustaine D, Iversen T, Kinne S, Kirkevåg A, Lamarque J-F, Lin G, Liu X, Luo G, Ma X, Penner JE, Rasch PJ, Seland Ø, Skeie RB, Stier P, Takemura T, Tsigaridis K, Wang Z, Xu L, Yu H, Yu F, Yoon J-H, Zhang K, Zhang H, Zhou C (2013). Radiative forcing of the direct aerosol effect from AeroCom Phase II simulations. Atmos. Chem. Phys.

[R19] Park RJ, Jacob DJ, Field BD, Yantosca RM, Chin M (2004). Natural and trans- boundary pollution influences on sulfatenitrate-ammonium aerosols in the United States: implications for policy. J. Geophys. Res.

[R20] Pouliot G, Denier van der Gon HAC, Kuenen J, Zhang J, Moran MD, Makar PA (2015). Analysis of the emission inventories and model-ready emission datasets of Europe and North America for phase 2 of the AQMEII project. Atmos. Environ.

[R21] Rao S, Mathur R, Hogrefe C, Keating T, Dentener F, Galmarini S (2012). Path Forward for the Air Quality Model Evaluation International Initiative (AQMEII). EM, Air And Waste Management Associations Magazine For Environmental Managers.

[R22] Schulz M, Chin M, Kinne S (2009). The Aerosol Model Comparison Project, AeroCom, Phase II: Clearing Up Diversity, IGAC Newsletter, No. 41.

[R23] Sekiya T, Sudo K (2014). Roles of transport and chemistry processes in global ozone change on interannual and multidecadal time scales. J. Geophys. Res.

[R24] Soares J, Sofiev M, Hakkarainen J (2015). Uncertainties of wild-land fires emission in AQMEII phase 2 case study. Atmos. Environ.

[R25] Sudo K, Akimoto H (2007). Global source attribution of tropospheric ozone: long-range transport from various source regions. J. Geophys. Res.

[R26] Sudo K, Takahashi M, Kurokawa J, Akimoto H (2002). CHASER: A global chemical model of the troposphere 1. Model description. J. Geophys. Res.

[R27] Dentener F, Keating T, Akimoto H, TF HTAP (Task Force Hemispheric Transport of Airpollution) (2010a). Part A, Ozone and Particulate Matter. Economic Commission for Europe.

[R28] Keating T, Zuber A, Dentener F, Seddon J, Travnikov O, Gusev A, Carmichael G, Parrish D, Grano D, TF HTAP (Task Force Hemispheric Transport of Airpollution) (2010b). Part D, Answers to Policy Relevant Science Questions.

[R29] Watanabe S, Hajima T, Sudo K, Nagashima T, Takemura T, Okajima H, Nozawa T, Kawase H, Abe M, Yokohata T, Ise T, Sato H, Kato E, Takata K, Emori S, Kawamiya M (2011). MIROC-ESM 2010: model description and basic results of CMIP5-20c3m experiments. Geosci. Model Dev.

